# Clinical utility of transthoracic echocardiography for screening abdominal aortic aneurysm: a prospective study in a Japanese population

**DOI:** 10.1186/s12947-016-0051-x

**Published:** 2016-02-12

**Authors:** Yoshihisa Matsumura, Michiko Wada, Daigo Hirakawa, Yuka Yasuoka, Norihito Morimoto, Hiroaki Takeuchi, Hiroaki Kitaoka, Kazumasa Orihashi, Tetsuro Sugiura

**Affiliations:** 1 Department of Laboratory Medicine, Kochi Medical School, Kochi University, Oko-cho, Nankoku-shi, Kochi 783-8505 Japan; 2 Clinical Laboratory, Kochi Medical School, Kochi University, Kochi, Japan; 3 Department of Cardiology, Neurology, and Aging Science, Kochi Medical School, Kochi University, Kochi, Japan; 4 Department of Cardiovascular Surgery, Kochi Medical School, Kochi University, Kochi, Japan

**Keywords:** Abdominal aortic aneurysm, Aortic root, Echocardiography, Screening

## Abstract

**Background:**

The aim of the present study was to evaluate the clinical utility of transthoracic echocardiography (TTE) for screening abdominal aortic aneurysm (AAA) and to identify important TTE indices associated with AAA in a Japanese population.

**Methods:**

We prospectively studied 1912 patients who were referred for TTE. AAA was defined as ≥ 30 mm in size.

**Results:**

The abdominal aorta was visualized in 95.1 % (1818/1912) by TTE. AAA was identified in 2.6 % (47/1818). The aortic root size was significantly larger in patients with AAA than those without (36.0 ± 4.1 vs. 31.7 ± 4.2 mm, *p* < 0.001). The aortic root size had a fair correlation with abdominal aortic size (r = 0.31, *p* < 0.001). The aortic root size of ≥ 34 mm was predictive of AAA by receiver operating characteristic curve analysis (area under the curve = 0.78, *p* < 0.001). Multiple logistic regression analysis revealed that aortic root size (Hazard ratio 1.23, *p* < 0.001) and age (Hazard ratio 1.05, *p* = 0.013) were the independent predictors of AAA.

**Conclusions:**

The feasibility of the abdominal aortic visualization during TTE was excellent. The aortic root size measured by TTE was the independent predictor of AAA. Screening for AAA during TTE appeared to be useful especially in the older patients with a large (≥34 mm) aortic root.

## Background

Abdominal aortic aneurysm (AAA), an abnormal focal dilation of the abdominal aorta, is a common and potentially life-threatening condition [1]. Only a half of patient with ruptured AAA can reach the hospital alive with an additional high operative mortality of 49 % [2]. In asymptomatic patients with AAA, elective repair of AAA is the most effective management to prevent the rupture of AAA with a low procedure mortality [3, 4]. Thus, early identification and elective repair of AAA is clinically important. Most of the patients with rupture have an undiscovered AAA [5], because AAA is mostly asymptomatic and occult on physical examination. Abdominal ultrasound, a fast and safe screening method to detect AAA with high sensitivity and specificity, is recommended for screening the patients who are at high risk of AAA [6]. Although screening for AAA during transthoracic echocardiography (TTE) has been reported to be clinically useful in Western populations [7–16], little is known about the important TTE indices associated with the presence of AAA [12, 14, 16]. Accordingly, the aim of this study was to evaluate the clinical utility of TTE for screening AAA and to identify important TTE indices associated with AAA in a Japanese population.

## Methods

### Study population

We prospectively examined 1912 consecutive patients who were referred for routine TTE from June 2013 to May 2014 at Kochi Medical School Hospital. Clinical data were obtained by chart reviews. The study was approved by the Ethics Committee on Human Research of the Kochi Medical School and all the patients gave written informed consent before the study. Patients with prior AAA repair or aortic dissection were excluded from the study.

### Echocardiography

Echocardiographic evaluation of the aorta was performed by 3 registered sonographers (M.W., D.H., and Y.Y.) in our echocardiographic laboratory. Routine transthoracic 2-dimensional, M-mode, and Doppler echocardiography were performed as recommended by the American Society of Echocardiography [17]. The aortic root size was measured at the level of the sinus of Valsalva from the parasternal long-axis view at the onset of the QRS complex. We used 4 echocardiographic equipments as follows: 1) Vivid7 (General Electric, Horten, Norway) with a 3S transducer (1.5 to 3.6 MHz variable-frequency), 2) iE33 (Philips, Andover, MA, USA) with an S5-1 transducer (1 to 5 MHz variable-frequency), 3) Vivid Q(General Electric, Horten, Norway) with a M4S transducer (1.5 to 3.6 MHz variable-frequency), 4) Prosound α 7 (Hitachi-Aloka Medical, Tokyo, Japan) with a UST-52105 transducer (3.5 to 5.0 MHz variable-frequency transducer).

In succession to routine TTE, the abdominal aorta was visualized in the supine position using the same cardiac transducer within a short period of time. First, longitudinal image of the abdominal aorta was visualized. Secondly, transverse image of the abdominal aorta was visualized. The abdominal aorta was scanned from the subcostal position and then traced distally as far as possible. Size of the abdominal aorta was measured using still-frame images and ‘on-line’ video calipers in diastole at the onset of the QRS complex at the longitudinal and transverse images. The maximal size of the abdominal aorta ≥ 30 mm in either anteroposterior or lateral size was defined as AAA. Clinical information was collected using the TTE requisition slip and the electrical recording system. In randomly selected 15 patients, we measured the additional examination time for screening AAA.

### Statistical analysis

Categorical variables were presented as total number and % of patients, and continuous variables were presented as means ± standard deviation. Clinical and TTE indices between the 2 groups were compared with chi-square test for categorical variables and Wilcoxon rank-sum test for continuous variables. Correlation between the aortic root and the abdominal aorta sizes was evaluated using linear regression analysis. Receiver operating characteristic curve analysis was used to determine the discriminating cutoff value for predicting AAA. Multiple logistic regression analysis was used to determine statistically significant variables associated with AAA. A *p* value < 0.05 was considered statistically significant. Data were statistically analyzed using the JMP version 11.0 software (SAS Institute Inc., Cary, NC, USA).

## Results

### Feasibility of AAA screening during TTE

Among 1912 patients, the abdominal aorta was visualized in 95.1 % (1818/1912) of the patients. The additional examination time for abdominal screening was less than a minute; the average time for abdominal aorta screening was 31.4 ± 12.3 (ranged from 13.0 to 53.0) seconds in randomly selected 15 patients. TTE was performed with Vivid7 in 36 %, with iE33 in 34 %, with Vivid Q in 17 %, and with Prosound α 7 in 13 % of the patients.

### Patient characteristics

We analyzed 1818 patients in whom adequate visualization of the abdominal aorta were obtained. Forty-one percent (746/1818) were inpatients and 49 % (897/1818) were men with a mean age of 67.4 ± 15.8 years. The clinical and echocardiographic diagnosis of the study patients were: ischemic heart disease (24 %), valvular disease (23 %), myocardial disease (22 %), arrhythmia (7 %), congenital heart disease (3 %), ventricular hypertrophy (3 %), pulmonary hypertension (2 %), cardiac mass (1 %), normal echocardiogram (12 %) and others (3 %). The mean size of the abdominal aorta was 17.2 ± 5.5 mm.

### Prevalence of AAA

AAA was identified in 2.6 % (47/1818). The prevalence of AAA was 3.9 % (35/897) in men and 1.3 % (12/921) in women (*p* < 0.001). The mean size of AAA was 43.6 ± 10.7 (30.0 to 90.0) mm, with 34 patients (72.3 %) having an aortic diameter ≥ 40 mm. Of the 47 patients with AAA, 29.7 % (14/47) underwent surgical repair or endovascular aortic stent graft insertion during 12 months following the TTE. AAA was previously known in 30 patients and unknown in 17 patients. Thirty-four of the 47 patients with AAA (72 %) had a prior or current history of smoking, 33 (70 %) had hypertension, and 26 (55 %) had ischemic heart disease.

### Clinical and TTE indices associated with the presence of AAA

Clinical and TTE indices in patients with and without AAA are shown in Table 1. The patients with AAA were significantly older than those without (77 ± 9 vs. 67 ± 16 years, *p* < 0.001). AAA was not found before the age of 54 years. AAA was significantly more frequent in male patients than female patients. The prevalence of AAA in male patients aged ≥ 55 years was 4.6 % (35/765).

**Table 1:** Comparison of clinical and echocardiographic indices

Variables	AAA	AAA	*p* value
	(+)	(-)	
Age (years)	77 ± 9	67 ± 16	<0.001
Men, n (%)	35(75 %)	862(49 %)	0.001
Aortic root (mm)	36.0 ± 4.1	31.7 ± 4.2	<0.001
Left atrium (mm)	37.3 ± 6.9	38.1 ± 7.7	0.664
Interventricular septal thickness (mm)	9.7 ± 1.4	9.6 ± 2.0	0.323
LV posterior wall thickness (mm)	9.7 ± 1.1	9.3 ± 1.5	0.018
LV end-diastolic dimension (mm)	47.8 ± 4.3	46.2 ± 6.1	0.010
LV end-systolic dimension (mm)	31.0 ± 5.5	28.8 ± 7.0	0.001
LV fractional shortening (%)	34.9 ± 7.8	37.8 ± 8.5	0.001
LV mass (g)	168.1 ± 41.2	156.0 ± 51.9	0.016
E (cm/sec)	59.6 ± 18.1	70.6 ± 23.8	0.001
A (cm/sec)	85.3 ± 25.2	79.6 ± 24.6	0.192
E/A	0.75 ± 0.37	0.93 ± 0.47	0.001
E deceleration time (msec)	247.1 ± 61.5	232.8 ± 65.8	0.109
E’ (cm/sec)	5.3 ± 1.8	6.6 ± 2.5	0.001
A’ (cm/sec)	9.5 ± 2.3	9.3 ± 2.3	0.960
S’ (cm/sec)	6.4 ± 1.8	7.0 ± 1.9	0.022
E/E’	12.2 ± 5.9	11.8 ± 5.6	0.568

Data are presented as mean ± SD or n (%). LV, left ventricular. E, Early diastolic LV inflow velocity; A, Late diastolic LV inflow velocity; E’, early diastolic septal mitral annular velocity; A’, late diastolic septal mitral annular velocity; S’, systolic septal mitral annular velocity; TTE = Transthoracic echocardiography; AAA = abdominal aortic aneurysm

There were significant differences in the aortic root size, left ventricular (LV) posterior wall thickness, LV end-diastolic dimension, LV end-systolic dimension, LV fractional shortening, LV mass, early diastolic LV inflow velocity, early diastolic LV inflow velocity/ late diastolic LV inflow velocity, early diastolic septal mitral annular velocity, and systolic septal mitral annular velocity between patients with and without AAA.

**Fig. 1:**
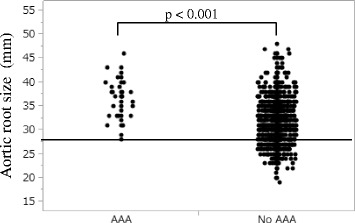
The aortic root size in patients with and without AAA. The aortic root size was significantly larger in patients with AAA than those without (36.0 ± 4.1 vs. 31.7 ± 4.2 mm, p < 0.001). All patients with AAA had the aortic root size of ≥ 28 mm. AAA = abdominal aortic aneurysm

**Fig. 2:**
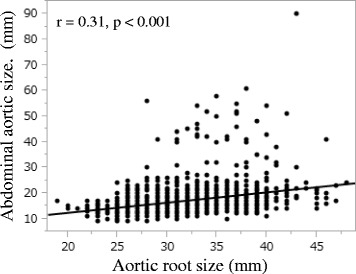
Correlation between the aortic root and the abdominal aorta sizes. The aortic root size correlated significantly with the abdominal aortic size (r = 0.31, *p* < 0.001)

The aortic root size measured by TTE was significantly larger in patients with AAA than those without (36.0 ± 4.1 vs. 31.7 ± 4.2 mm, *p* < 0.001) (Fig. 1). All patients with AAA had aortic root size of ≥ 28 mm. The aortic root size correlated significantly with the abdominal aortic size (r = 0.31, *p* < 0.001) (Fig. 2). The aortic root size of ≥ 34 mm was predictive of AAA by receiver operating characteristic curve analysis (area under the curve = 0.78, sensitivity: 70 %, specificity: 70 %, *p* < 0.001). Multiple logistic regression analysis using 12 variables revealed that aortic root size (Hazard ratio 1.23, *p* < 0.001) and age (Hazard ratio 1.05, *p* = 0.013) were the independent predictors of AAA (Table 2).

**Table 2:** Multiple logistic regression analysis for predicting AAA

Variables	Hazard ratio	95 % CI	*p* value
Age (years)	1.05	1.01-1.09	0.013
Men, n (%)	1.27	0.57-2.90	0.562
Aortic root (mm)	1.23	1.13-1.33	<0.001
LV posterior wall thickness (mm)	1.55	0.93-2.67	0.10
LV end-diastolic dimension (mm)	1.23	0.98-1.53	0.067
LV end-systolic dimension (mm)	0.90	0.75-1.14	0.352
LV fractional shortening (%)	0.94	0.86-1.06	0.242
LV mass (g)	0.98	0.95-0.99	0.054
E (cm/sec)	1.00	0.97-1.02	0.741
E/A	0.68	0.17-1.70	0.489
E’ (cm/sec)	0.92	0.69-1.18	0.558
S’ (cm/sec)	0.86	0.66-1.10	0.235

Data are presented as mean ± SD or n (%). LV, left ventricular. E, Early diastolic LV inflow velocity; A, Late diastolic LV inflow velocity; E’, early diastolic septal mitral annular velocity; A’, late diastolic septal mitral annular velocity; S’, systolic septal mitral annular velocity; AAA = abdominal aortic aneurysm; CI, confidence interval

## Discussion

The present study had 3 major findings. First, the feasibility of the abdominal aortic visualization during TTE was excellent (95.1 %). Second, the prevalence of AAA during TTE was 2.6 %. Third, the aortic root size measured by TTE was the independent predictor of AAA.

Reynolds T et al. firstly reported in 1990 that evaluation of the abdominal aorta should be carried out during a routine TTE examination [18]. Afterwards, other authors have reported usefulness of TTE for screening AAA [7–16], because of excellent feasibility and relatively high prevalence of AAA.

The success rate of abdominal aortic imaging during TTE has been reported to range from 79 to 96 % with an additional time range from 0.5 to 8 min for examination [7–16]. In the present study, we used the same cardiac transducer, so that no additional equipment was required. Moreover, an additional examination time for abdominal visualization was less than a minute in randomly selected 15 patients. These results suggest that screening of the abdominal aorta during TTE is feasible with minimal additional time and cost compared to separate abdominal ultrasound examination.

The prevalence of AAA during TTE has been reported to range from 2.2 % to 6.5 % in Western countries [7–16], whereas the prevalence of AAA using abdominal ultrasound ranged from 3.0 % to 8.0 % from population screening surveys in Western countries [19]. Therefore, previous studies have demonstrated that screening for AAA during TTE is clinically useful [7–16]. However, Seelig MH et al. demonstrated that TTE performed in a highly selected cardiac patient group in a tertiary referral center is not a useful tool for screening clinically unsuspected AAA [20], because the prevalence of AAA was low (0.8 %) and only 16 % of the patients with AAA underwent surgical repair following TTE. In Korean population [21], detection rate of AAA was 0.5 % during TTE indicating that the prevalence of AAA in Asia is lower than that in Western countries. Despite the low prevalence of AAA, a routine examination of the abdominal aorta during TTE appeared to be an effective preventive strategy for life-threatening but asymptomatic AAA, because 26 % of the patients with AAA underwent surgical repair or endovascular aortic stent graft insertion following the TTE. In the present study, the prevalence of AAA during TTE was 2.6 %. Moreover, 30 % of the patients of the present study with AAA underwent aortic repair following the TTE. These results suggest that screening for AAA during TTE also appeared to be clinically useful in a Japanese population.

Among the TTE indices associated with the presence of AAA, LV hypertrophy, LV dilatation, or low LV fractional shortening are likely relate to hypertension; 70 % of the patients with AAA had hypertension. Although Bekkers SC et al. reported that patients with AAA had dilated ascending aorta [12], little is known about the clinical importance of the aortic root size in association with AAA [14, 22]. Recently, Aboyans et al. have reported that the ascending aorta is larger in patients with AAA than those without by univariate analysis [16], though it is unknown about the exact position of measurement of the ascending aorta. Agricola E et al. have also demonstrated a high prevalence of dilatation/aneurysm of the ascending aorta and the aortic arch in patients with AAA evaluated by TTE [23]. These studies suggest a significant correlation between dilatation of the ascending aorta and the arch and AAA. However, discriminating cutoff value of the ascending aortic size for predicting AAA was not determined. Multivariate analysis was also not used to determine the significance of the ascending aortic size in association with AAA. Thus, it still remains unknown whether the ascending aortic size is important TTE indices for screening AAA.

In contrast, we measured the aortic root size at the level of the sinus of Valsalva. The aortic root size of ≥ 34 mm was predictive of AAA by receiver operating characteristic curve analysis. The aortic root size was the independent TTE index associated with the presence of AAA. As the common risk factors for AAA and atherosclerosis may lead to the aortic root dilatation, considering the fact that aortic root size and age were independent predictors of AAA, our study indicate that screening for AAA during TTE can be useful especially in the older patients with a large (≥34 mm) aortic root size. Although screening for AAA during TTE has been reported to be useful [7–16], the screening limiting to the older patients with a large (≥34 mm) aortic root size can be clinically efficient.

The present study has several limitations. First, the incidence of AAA depends on patient selection. However, the prevalence of AAA of 2.6 % in the present study was not significantly different of that reported in Western countries (2.2 % to 6.5 %). Second, AAA was incidentally discovered before the present study at least in 18 patients (60 %) as the result of other abdominal imaging studies obtained to evaluate an unrelated condition such as spine magnetic resonance imaging, computed tomography, or abdominal ultrasound, though the reasons for diagnosis of AAA were not available in the other 12 patients. The increasing use of imaging modalities has revealed asymptomatic and previously undiagnosed AAA. Therefore, we did not exclude patients with previously known AAA. Third, the atherosclerotic risk factors in patients with AAA were only available concerning a prior or current history of smoking, hypertension, and ischemic heart disease in the present study. Moreover, the atherosclerotic risk factors in patients without AAA were not available. The lack of information on the prevalence of traditional risk factors for atherosclerosis in patients with and without AAA does not allow stratifying the patients more likely to have AAA, according to atherosclerotic risk profile. Fourth, early detection of small AAA during TTE could cause needless disease labeling and anxiety resulting in psychological harm. Further studies are required to verify the clinical usefulness, cost-effectiveness, and psychological harm of AAA screening during TTE in different healthcare settings.

## Conclusions

Visualization of the abdominal aorta during TTE is feasible with little additional time and cost. The aortic root size was the important TTE index associated with the presence of AAA. Screening for AAA during TTE was especially useful in the older patients with a large (≥34 mm) aortic root size.

## Abbreviations

AAA: Abdominal aortic aneurysm
TTE: Transthoracic echocardiography
LV: Left ventricular
